# Surgical Management of a Three-Month-Old Mal-United Dubberley Type 2A Distal Humerus Fracture: A Case Report

**DOI:** 10.7759/cureus.58865

**Published:** 2024-04-23

**Authors:** Darshan H Sharma, Sushil Mankar, Rahul H Sakhare, Vismay V Harkare

**Affiliations:** 1 Orthopedics and Trauma, N.K.P Salve Institute of Medical Sciences & Research Center and Lata Mangeshkar Hospital, Nagpur, IND; 2 Orthopedics and Traumatology, N.K.P Salve Institute of Medical Sciences & Research Center and Lata Mangeshkar Hospital, Nagpur, IND; 3 Orthopedics, N.K.P Salve Institute of Medical Sciences & Research Center and Lata Mangeshkar Hospital, Nagpur, IND

**Keywords:** dubberley classification, anterior approach for elbow, mal-united fracture, trochlea fracture, capitellum fracture

## Abstract

Fractures of the capitellum and trochlea are not common in orthopedic trauma and pose certain difficulties to address and manage. On primary x-rays, these fractures are commonly missed, and patients may be treated inadequately resulting in a restricted range of motion. The current case report presents the surgical outcome and challenges faced while managing a 30-year-old male patient with a mal-united capitellum, trochlea, and lateral condyle of humerus fracture. The patient had come with complaints of a restricted range of motion in his dominant hand which affected his livelihood. After undergoing adequate investigations, the patient was posted for an open reduction and internal fixation. The approach used for the procedure and the challenges faced during the surgery have been elaborated in the case report. The patient had shown an increase in the range of motion which was maintained at six- and nine-month follow-ups. Thus, it states that patients with trochlea and capitellum fractures presenting late and having a restricted range of motion can be managed adequately with good outcomes after proper planning.

## Introduction

About 6% of distal humerus fractures and 1% of all elbow fractures are fractures of the capitellum and trochlea are very rare [1]. Isolated fractures of the trochlea are even more rare [2]. The capitellum is spherical in nature made by the anterior and inferior surface of the distal humerus and fracture of which the elbow joint unstable [3]. Women are more prone to shear fractures of the distal humerus which can be attributed to recurvatum, poor bone stock, and cubitus valgus. A low-energy trauma due to a fall over an outstretched hand or while reduction of the postero-lateral elbow dislocation can also lead to this kind of fracture [4]. These fractures can also be caused by direct transmission of force via the radial head and a shearing and/or axial force to the distal humerus [5]. The diagnosis and treatment of the osteochondral fracture of the distal part of the humerus are not easy. Complex anatomy, difficult exposure, fragile bone stock, and pattern of fracture are the various difficulties encountered by treating surgeons [6]. The most frequent fracture pattern of the fracture of the capitellum is the osteochondral fragment [7]. Failure to reduce anatomically the capitellum and trochlea fracture can alter the range of motion and the stability of the elbow provided by the trochlea [8]. Managing the fracture conservatively which was a previously accepted treatment protocol for these fractures has often led to unsatisfactory results [6]. Closed reduction, fragment excision, arthroscopic assisted internal fixation and open reduction and internal fixation are the various treatment options available for the management of fracture trochlea and capitellum [1]. McKee described the partial articular fractures of the trochlea and capitellum and gave a classification system [7]. Dubberley gave a classification system focusing on the treatment as well as the outcome of coronal fractures of the capitellum and it considers the involvement of the trochlea and the posterior communication [3].

Dubberley’s classification system aids with pre-operative planning and deciding surgical tactics. With the increasing type of fracture complexity, the need for exposure to address the fracture medially is required. It also takes into account the posterior comminution. They classified the fracture depending on the involvement of the parts of the distal humerus namely into three types with type 1 including fracture of capitellum primarily with or without the involvement of lateral trochlear ridge. Type 2 includes the trochlea and capitellum as single pieces and type 3 consists of both the trochlea and capitellum as separate fragments. Depending on the absence or presence of posterior condylar comminution these fractures were further characterized into A and B [9]. Fractures of the humerus lateral condyle represent a unique and relatively uncommon (<2%) subset of elbow injuries, particularly in the elderly population [10]. The lateral condyle, a critical bony prominence, plays a crucial role in maintaining joint stability and facilitating the intricate movements of the upper extremity. Injuries to this region can significantly impact the biomechanics of the elbow, potentially leading to long-term functional impairment if not appropriately addressed [11].

## Case presentation

Patient information

A young 30-year-old male right-hand dominant, electrician by occupation had a history of trauma due to a fall from height. The patient had gone to a quack, where he was managed with some folk treatment. The patient presented after three months to us with complaints of a restricted range of motion. The patient had also difficulty performing his daily activities and had difficulty earning his livelihood due to the complaints.

Clinical findings

On examination, the patient had 30 degrees of flexion deformity in the elbow joint. The patient did not complain of any tenderness at the elbow joint. The patient presented with a restricted range of motion which was from 30 degrees to 60 degrees of elbow flexion, further movement of the elbow was restricted but not painful.

Timeline

The patient presented with a restricted elbow range of motion in the dominant hand which led to difficulty for the patient to earn a livelihood. The patient had a history of trauma three months back which was managed by a quack. A previous x-ray might not have been done as no x-ray was available with the patient. The patient had undergone investigations namely an x-ray and CT scan of the elbow joint. Generalized investigations of the patient were performed and were found to be normal. Meticulous planning was done for the patient and surgery was performed. The patient had an improved range of motion from 10 degrees to 80 degrees of elbow flexion. The patient was followed up for one year and on follow-up, the patient had an improved range of movement of the right elbow and the patient was able to get back to his livelihood.

Diagnostic assessment and interpretation

**Figure 1:**
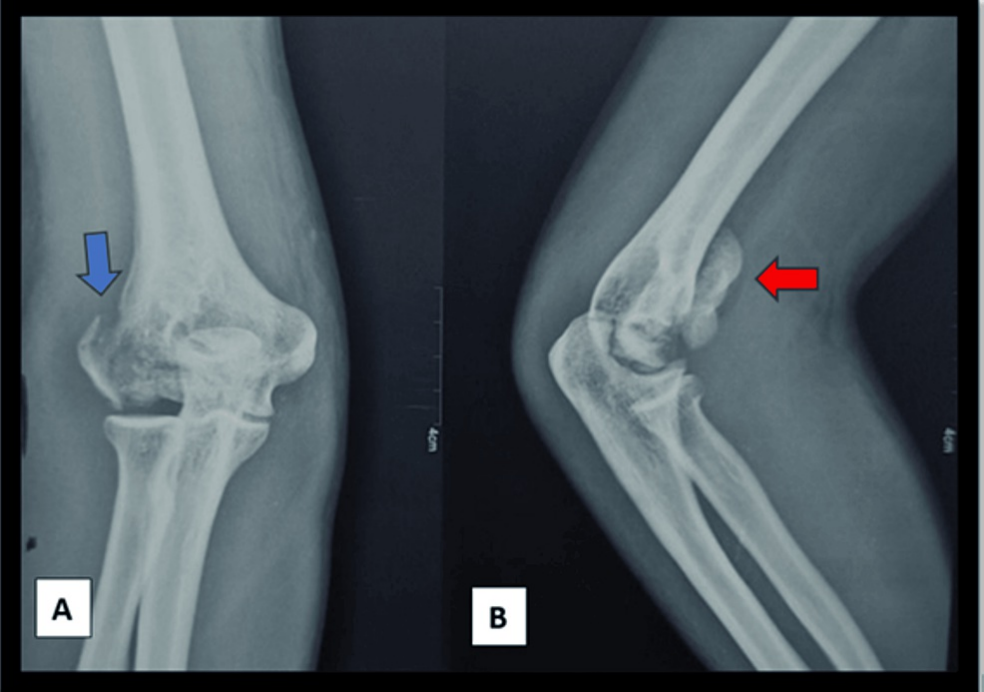
Preoperative radiological image (A) Anteroposterior x-ray view showing lateral condyle of humerus fracture (blue arrow). (B) Lateral x-ray showing capitellum and trochlea fracture with classical double rim sign (red arrow).

**Figure 2:**
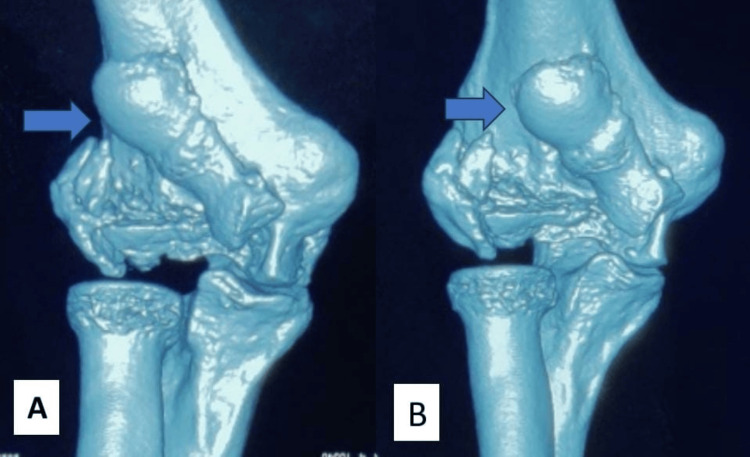
CT images (A) and (B) showing fracture and proximal migration of capitellum and trochlea (blue arrows).

For diagnosis of the fracture, an elbow anteroposterior and lateral view x-rays were obtained. The x-ray of the elbow joint showed a fracture of the lateral condyle of the humerus with a coronal shear and migration of the capitellum and trochlea proximally. The lateral condyle of the humerus was also fractured but appeared to be malunited on the anteroposterior x-ray (Figure 1A). The x-ray showed a classical double rim sign in the lateral view which was suggestive of both capitellum and trochlea fracture (Figure 1B). A proper anteroposterior view of the elbow joint could not be obtained as the elbow could not be extended beyond 30 degrees of flexion. A CT scan of the elbow was also performed to get a clear idea of the fracture morphology. CT scan confirmed the coronal shear fracture of trochlea and capitellum as one fragment that was migrated proximally and a minimally displaced fracture of the lateral condyle of humerus fracture (Figures 2A, 2B). Based on the x-ray and CT images a diagnosis of coronal shear fracture of capitellum and trochlea with a minimally displaced fracture of the lateral condyle was made and was classified as type 2A Dubberley.

Intervention

**Figure 3:**
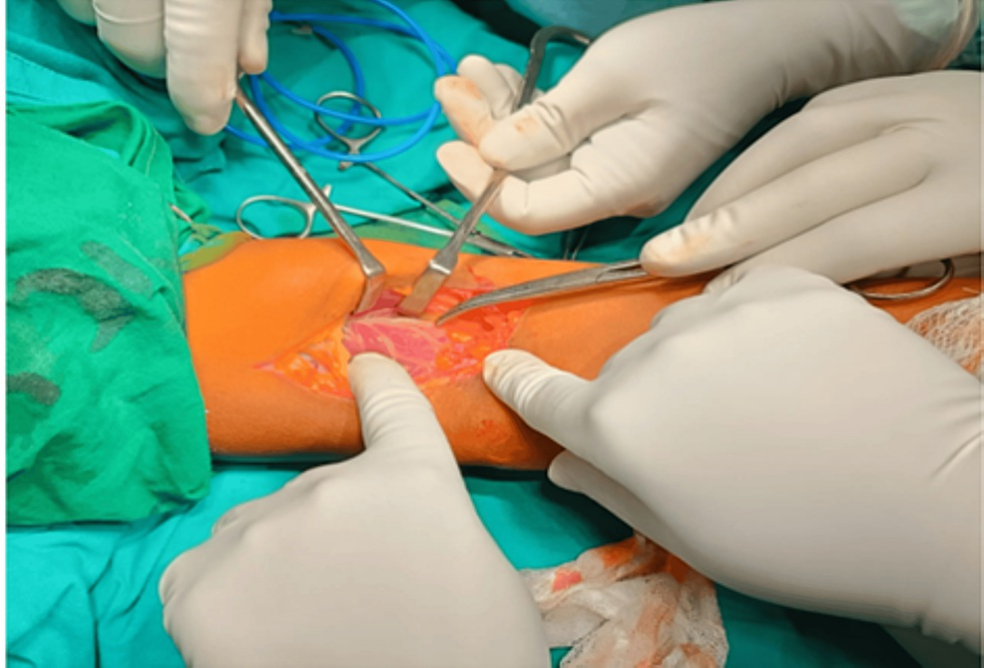
Subcutaneous dissection done after giving a Lazy S-shaped incision over anterior aspect of the elbow

**Figure 4:**
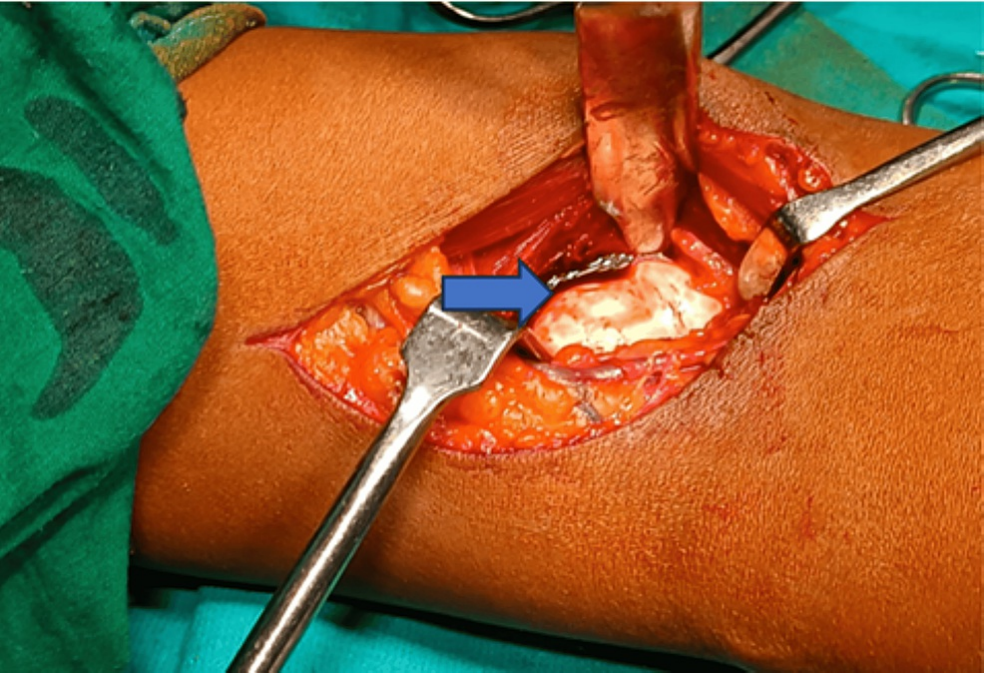
Surgical exposure of fracture site showing the proximal migration of the fracture fragment (blue arrow)

**Figure 5:**
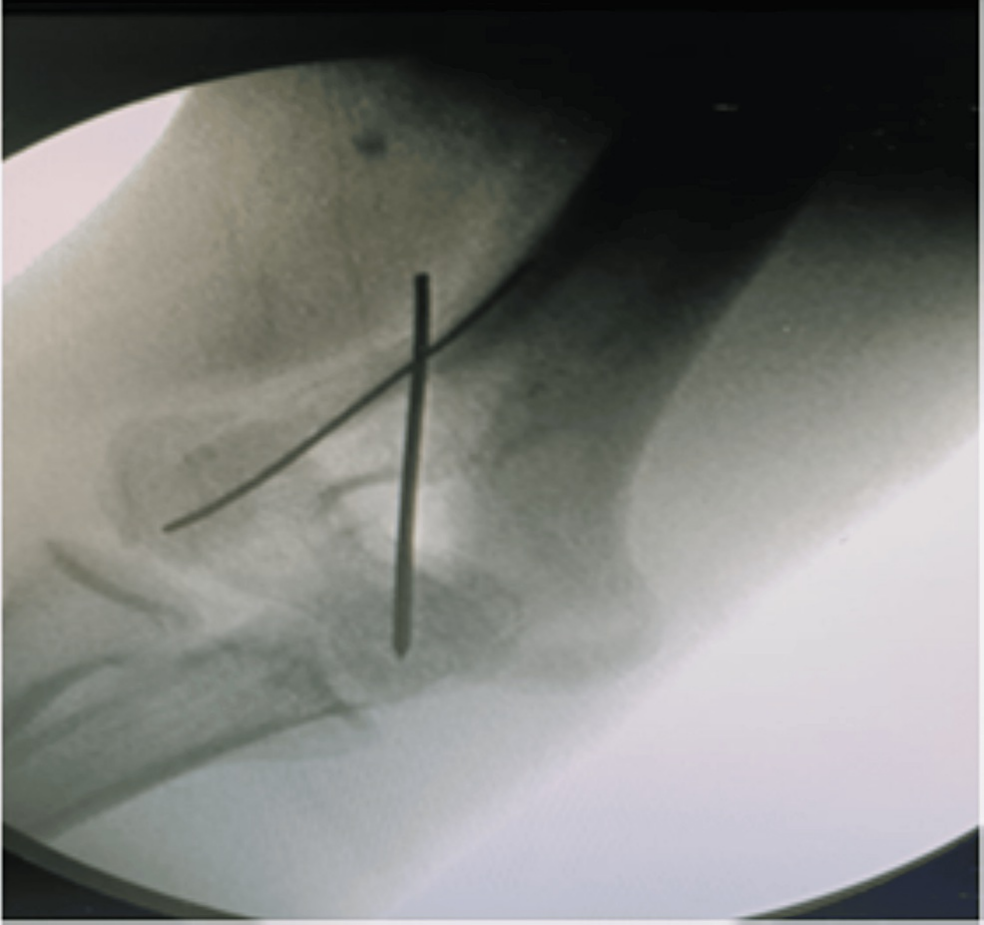
Intra-operative C-arm image showing the use of K-wire fixation of fracture fragments

The patient was posted for an open reduction and internal fixation of the capitellum and trochlea fracture. The primary plan was to fix the fracture using a compression headless screw. A backup plan and implants were kept ready for the lateral humeral condyle fracture in case the fracture was not united. An anterior approach with a lazy S incision was planned for the patient as it would provide easy access to the fragments of the trochlea and capitellum and fixation would be easy (Figure 3). A meticulous dissection was carried out. The fracture fragments of the trochlea and capitellum were identified which were found migrated proximally and mal-united there (Figure 4). A careful osteotomy was done around the fracture fragment and the fragment as a whole was mobilized to achieve reduction. A temporary k-wire was passed to hold the fracture fragment and reduction was checked under the c-arm, which was found to be adequate, joint congruency was also found to be adequate (Figure 5) and fixation was done with Herbert Screw. The lateral condyle of the humerus was found to be united hence was not addressed. An above elbow slab was given to the patient for three weeks in 20 degrees of flexion to facilitate healing.

Follow-up and outcome

**Figure 6:**
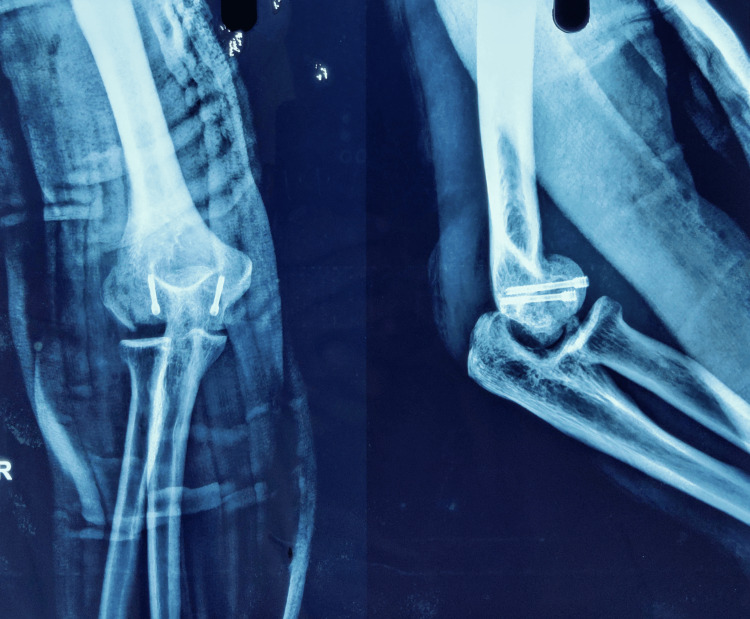
Three-week post-operative x-ray with above elbow slab in situ

**Figure 7:**
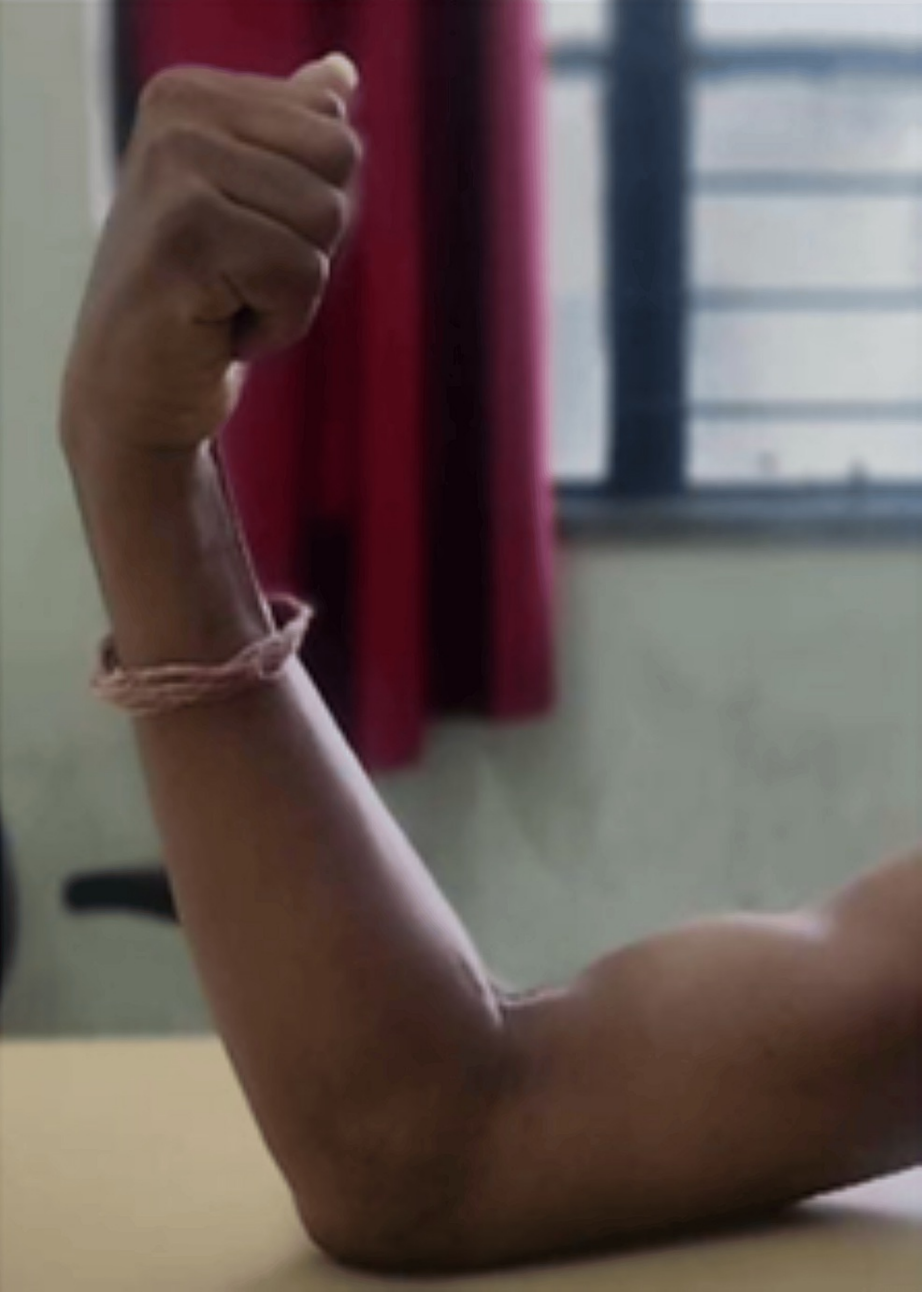
Flexion of 80 degrees achieved at sixth-month follow-up

The above elbow slab was removed at three weeks (Figure 6) and the patient was advised a range of motion exercises for the elbow. The patient post-operatively had a range of motion from 10 degrees to 80 degrees (Figure 7). The patient was advised physiotherapy and passive range of motion exercises. At six months follow-up, the patient had an improvement in range of motion, and he was able to get back to his livelihood (Video 1). To further assess the outcome, we evaluated the elbow performance using the Mayo Elbow performance score [12]. We found a score of 95, signifying excellent elbow performance.

**Video 1:** Six months old post-operative elbow range of motion Post-operative six months elbow range of motion showing flexion, extension, supination, and pronation movement.

## Discussion

A coronal shear pattern fractures of the distal humerus usually involve the capitellum alone, but it may sometimes extend medially to involve the trochlea [1]. Other soft tissue injuries around the elbow joint associated with this injury lead to joint instability. Radial head fracture or lateral collateral ligament injury has a high association with these kinds of fractures [1]. Various treatment modalities are available for the management of the distal humerus fracture including excision of the fragment, arthroscopic assisted reduction and internal fixation, closed reduction, etc. When internal fixation cannot be achieved Arthroplasty can be considered [1].

Our particular patient had a restricted range of motion due to trauma to the right elbow which was his dominant hand and resulted in the loss of his livelihood. We attempted open reduction and internal fixation of the shear fracture fragment using a headless compression screw.

McKee et al. in 1996 were the first authors to describe the importance of recognizing the trochlear fracture pattern to plan pre-operatively. They identified the shear fracture pattern of the articular surface and described the fracture of the capitellum and trochlea showing double arc signs on radiographs [7].

Dubberley et al. in 2006 found the current classification systems for the distal humerus to be descriptive and not helpful in directing the treatment. They reported their results in 38 patients with distal humerus fracture all addressed with a posterior approach and found good results in terms of range of motion and mayo elbow score. They also reported a complication of non-union and fractures with posterior comminution and stated the requirement of elbow arthroplasty. They also gave a classification system based on radiographs [9].

We classified the fracture based on the Dubberley classification as it gives a better description and better guidance for further approach. Imatani et al. in 2001 mentioned the importance of trochlea in the stability of elbow joints due to its anatomy. They addressed the patients with distal humerus fractures and used an anterolateral approach. They found the approach to be better than the posterior approach for coronal fractures of the distal humerus [8].

Ballersteros-Betancourt et al. in 2020 [13] stated their study in eight patients of capitellum and trochlea fractures managed with open reduction and internal fixation with an anterior limited approach to the elbow (ALAE) and found the results to be better for isolated capitellar fractures. They also promoted the use of ALAE as a better option for the management of capitellar and trochlea fractures. In our present case scenario, a similar approach was used for the management of capitellum and trochlea fractures. We found the approach to be very helpful in addressing the fracture.

Yoshida et al. in 2021 [6] mentioned the association of fracture capitellum and lateral epicondyle fracture and found the outcome to be worse in patients with fracture extension into trochlea and posterior comminution. They used the MEPI score to assess the outcome and found it to be significantly affected by posterior comminution. Vyas et al. in 2016 [14] performed a study with 16 capitellum fractures using anterolateral approach and headless compression screw and found good results with a mean range of flexion of 132 degrees. They found the anterolateral approach to be beneficial in terms of preservation of the extensor origin and assistance in fracture fixation. Preserving the posterior blood supply is also a benefit of the approach. Shergold et al. in 2021 [15] conducted a study among 45 patients having coronal fractures of the distal humerus which were classified according to the modified Dubberley classification. They found great results with an average flexion arc of 125 degrees. For management of type B fractures they also recommended a combined compression plate and screw.

## Conclusions

Intra-articular distal humerus fractures involving the capitellum and trochlea are rare. This case demonstrates that using an anterior approach for elbow three-month-old Dubberley type 2A can be managed with a good range of motion from 10 to 80 degrees of flexion. Other studies report a huge improvement in the range of motion. A similar kind of range of motion was not appreciated in our case which can be attributed to the three-month-old history of trauma leading to joint stiffness. The patient was advised physiotherapy and reported an improvement in range of motion. More importantly, the patient was able to continue his daily livelihood.
